# Moderating Effect of Help-Seeking in the Relationship between Religiosity and Dispositional Gratitude among Polish Homeless Adults: A Brief Report

**DOI:** 10.3390/ijerph19031045

**Published:** 2022-01-18

**Authors:** Małgorzata Szcześniak, Katarzyna Szmuc, Barbara Tytonik, Anna Czaprowska, Mariia Ivanytska, Agnieszka Malinowska

**Affiliations:** Institute of Psychology, University of Szczecin, 71-017 Szczecin, Poland; katarzynaszmuc7@gmail.com (K.S.); barbara.tytonik@op.pl (B.T.); czapranna1a@gmail.com (A.C.); m.ivanytska.usz@gmail.com (M.I.); agnieszka.malinowska@usz.edu.pl (A.M.)

**Keywords:** help-seeking, religiosity, dispositional gratitude, homeless, adults

## Abstract

Although empirical reports draw attention to the pathological aspects of the functioning of the homeless, recent studies show the benefits related to the elevating roles of different positive phenomena in coping with difficulties for this group of people. The main goal was to verify whether there is a direct relationship between religiosity and gratitude among the homeless, and whether this association is moderated by the reported help-seeking since both religiosity and gratitude seem to play an important role in homeless people’s lives. In total, 189 homeless persons participated in the study. Their mean age was *M* = 56.55 (*SD* = 12.39; range = 27–86). Most respondents were men (*n* = 119; 63%). The Scale of Religious Attitude Intensity and the Gratitude Questionnaire were used. The outcomes presented a statistically significant positive correlation between religious attitude and gratitude (*r* = 0.326***, *p* = 0.001). Help-seeking played a moderatory role in this relationship. Therefore, it can be assumed that the relationship between religiosity attitude intensity and dispositional gratitude is stronger when homeless persons seek specific help from other people or institutions compared to when they do not look for assistance. Homeless people, overcoming their limitations by actively asking for help, can strengthen their bonds with God (faith, religiosity) and with others (dispositional gratitude).

## 1. Introduction

Homelessness is widely considered as an individual [1,2,3], social [3,4,5,6], economic [7,8], and political [9] problem that affects the functioning of both individuals and whole of societies. Gu et al. [10] report that more than 150 million people worldwide are homeless. A nationwide survey carried out in 2019 at the initiative of the Ministry of Family, Labor and Social Policy showed that there were 30,330 homeless people in Poland, of which 82.1% were men, 14.6% women and 3.3% children [11]. Most of them (24,323) were institutionalized and concentrated in urban regions [9].

There is not a universally recognized and consistent definition of homelessness [12,13,14]. In a narrow sense, a homeless person is regarded as “someone living on the streets without shelter” ([15], pp. 717–726) or having a primary nighttime residence provided by an institution that serves this purpose [16]. More broadly, people who do not live in conventional, adequate, or secure accommodation and who are without economic and social support are categorized as homeless. Edgar et al. [17] enumerated three domains of exclusion that characterized homeless persons. The physical domain concerned a suitable dwelling, the social domain involved privacy and maintaining social relations, and the legal domain pertained to legal title to private space.

Different studies [18,19,20,21,22,23,24] point to multiple causes of homelessness related mainly to socio-economic conditions (e.g., social policy, unemployment, debt), political components (e.g., cost-cutting in the welfare system, increased migration), social factors (e.g., loss of support of family or friends, domestic violence) and personal characteristics (e.g., cognitive disabilities, alcoholism and other forms of addiction, physical disabilities, poor health).

Although a significant portion of the literature and empirical reports draw attention to the pathological aspects of the functioning of the homeless [25], recent studies show the benefits related to the elevating roles of different positive phenomena in coping with difficulties for this group of people. For example, there is some evidence that helping the homeless to recognize their personal strengths [25,26] and respecting their dignity [27] may encourage them to get out of homelessness. Moreover, for some homeless people, verbalizing their own history, sharing difficulties and unpleasant emotions becomes a turning point in the further process of becoming independent [28]. Therefore, in light of the theoretical and practical potential in conducting research on such constructive aspects, we aimed to verify whether there is a direct relationship between religiosity and gratitude among homeless persons, and whether this association is moderated by the reported help-seeking. The rationale for selecting these specific variables was that both religiosity and gratitude seem to play an important role in homeless people’s lives.

### 1.1. Religiosity and Homelessness

Comprehensive research in the general population shows in large part that different dimensions of religiosity are associated with various facets of psychological well-being [29] although this relationship has also been found to not be always linear (higher religiosity → higher well-being) [30]. In several studies, trust in God [31], intrinsic religiosity [32], engagement in religious activities [33], religious meaning [34,35], and religious coping [36] are positively related to life satisfaction. Moreover, various researchers suggest that religiosity has a significant impact on gratitude. Prayer frequency [37], commitment to God [38], religious beliefs [39] and daily spiritual experiences [40] correlate positively with grateful dispositions.

However, little is known about religiosity [29,41,42] in the homeless population. Although Testoni et al. [43] point out that religiosity cannot be considered a factor preventative of addiction or suicidal thoughts and Longo et al. [44] suggest that religiosity may even play a harmful role and be a risk factor, some researchers [45,46,47] observe that participation in religious or spiritual practices may have an important positive effect on the lives of the homeless. At this point, it is important to mention that religiosity and spirituality, although closely related [48], are complex and distinct constructs [48,49,50]. Religiosity is typically considered a set of beliefs or rituals associated with participation in public settings [50] that allow proximity to a transcendent or theistic power [48]. In contrast, spirituality is conceptualized by some authors as a broader notion [51] that reflects the whole of the experiences and emotions connected to a personal search for comprehension, love, purpose, optimism, and self-fulfillment, and not to the adherence to community or religious practices [51]. Homeless people who declare higher levels of religiousness and spirituality or who attend religious services score lower on measures of suicidal ideation [52], have better mental health [53], feel they live a higher quality of life [54], present lower rates of substance abuse [46,55], are more resilient [56], hold low deviant beliefs [42], cope better with stress [57], adversity [58], and the strains resulting from homelessness [59,60] and are more likely to leave addiction behind [61]. Private and public forms of religiosity seem to give the homeless a sense of meaning [46] because the function of “meaning building” is generally assigned to religion [62]. Likewise, connectedness to the divine may provide them with other psychological benefits [45]. According to Szocik and Van Eyghen [35], religious beliefs provide sense and value to life. Many institutional shelters integrate religious practices into their programs to help the homeless handle their problems [63].

### 1.2. Gratitude and Homelessness

Nowadays, research on the topic of gratitude among the homeless is scarce. This may be due to the fact that a grateful disposition is difficult to preserve when one is deprived of basic needs [64] and does not have a safe place of refuge, family support or decent work [25]. Moreover, homeless persons are often perceived as “entitled rights-holders, not grateful supplicants” ([65], p. 805). They are widely considered a serious problem [5], a threat to the social order [66], passive and dependent [67] and suffering from the syndrome of learned helplessness [68].

However, some qualitative and quantitative studies report examples of gratitude among people experiencing homelessness. Motives for being appreciative and grateful are various: services and care provided for life-threatening illness [69,70], assistance [65] and time dedicated by the volunteers [71], support and help [72], a bed in a shelter [73], information and knowledge [74,75] and opportunities to learn [72]. Moxley and Washington [75] noted that some the homeless appreciate their experience of being without a home or economic security, regarding it as a personal journey. A recent study [76] conducted among youths living in emergency shelters showed that although they rarely reported gratitude in comparison to other VIA character strengths, they were grateful to the facilitators for their 10-week intervention. In another study, Rew and colleagues [77] acknowledged that gratitude together with hope and optimism explain life satisfaction, even after controlling for other variables. They also observed an inverse association between length of homelessness and gratitude. An interesting outcome was reported by Booth et al. [78]. In-depth interviews with charitable food services recipients showed that homeless people are grateful for any food but are dismayed when it is of poor quality (e.g., unhealthy) or lacks variety. In their exploratory study carried out with the participation of former runaway and homeless youths, Williams et al. [74] noted that these young people were grateful for gaining new knowledge thanks to their experiences, accepted healing relationships, found forgiveness and grew from “their brushes with death.” Lastly, people with histories of homelessness are grateful for safe homes [79].

### 1.3. Religiosity, Gratitude and Help-Seeking among the Homeless

As far as we know, no research into the relationship between religiosity and gratitude among the homeless has been carried out. However, there are some theoretical and empirical premises which make it possible to argue that such a relationship exists and may be moderated by the experience of seeking help. Some studies conducted among the general population present positive relationships between gratitude and having a religious orientation [80], conventional religious practices [81], spiritual self-transcendence [81], religious comfort [31], religious friends [82] and prayer [37].

According to Krause [83], religion may have a significant impact on feelings of gratitude. More specifically, it seems that believers both of monotheistic religions and Eastern traditions find numerous references in the scriptures and prayers to the necessity of being grateful [84,85]. For example, Judaism, Christianity, Islam, and Buddhism focus on gratitude as an important virtue which is a pathway to a good life [84,86]. Moreover, religion promotes gratefulness and other human virtues [87]. There is also some evidence that religious practices serve to express gratitude for life [88]. Emmons and Kneezel [81] observed that people who relied on God in difficult situations, whether trying to solve their problems themselves or entrusting them to God, tended to declare higher gratitude than people who did not count on divinity. Since homeless people resemble the general population as regards their religious beliefs [89] and religiosity is relevant to the well-being of the general population [29], it is possible to pursue similar kinds of research among the homeless.

With respect to both variables in the context of homelessness, religiosity and spirituality are considered by some homeless as sources of strength in their personal journeys which help them to understand, appreciate, and be grateful for their own life experiences [75]. Lovett and Weisz [61], after using qualitative interviews to analyze the role of religiosity in the functioning of homeless individuals, showed that building a positive relationship with God led to constructive changes in feelings of gratitude. Smith-Barusch ([90], p. 138) shed light on the influence of religiosity on disadvantaged women living in homeless shelters, reporting their gratitude for “life, good fortune, help in times of trouble, and material goods.” Moreover, God was considered by the participants of this study as the origin of all benefits.

According to Schieman and Bierman [91], religiosity through its “meaning-making function” has a positive impact on gratitude. Roemer [92] reported that in some cultures, gratitude has important value in religious services and rituals. These considerations bring an understanding of why homeless persons may experience gratitude. When people perceive their problems or life conditions as part of God’s project and see God as someone who wants to strengthen and help them grow, they more easily feel gratitude [93].

Another important aspect of the relationship between religiosity and gratitude among people experiencing homelessness may be the aspect of seeking help which consists of seeking assistance to solve a difficult situation or problem [94]. Krause [83] observed that there is a general belief that God helps people to overcome adversities by “using” the kindness and commitment of others. If religion assists people in their struggles with concrete forms of aid, they might be encouraged to ask for help and be grateful for the support received. In this context, the concept of “religious capital” seems important because it becomes a source of meaning when used in difficult situations [95]. Considering the current scientific achievements related to the subject of the homeless, their religiosity and gratitude, we assumed that:

**Hypothesis** **1** **(H1).** *A religious attitude correlates positively with gratitude among homeless persons*.

**Hypothesis** **2** **(H2).** *The level of gratitude resulting from the intensity of religious attitude is significantly different at different levels of seeking help by homeless persons*.

**Figure 1:**
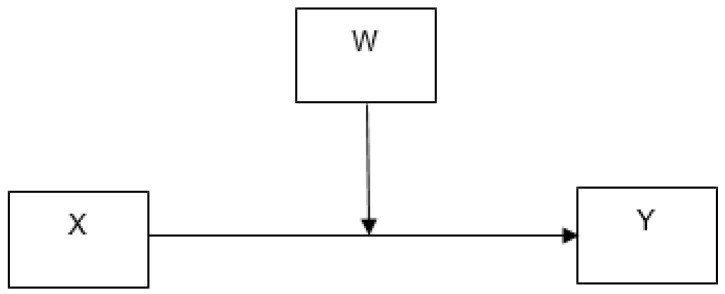
Conceptual model with a single moderator.

In this study, religious attitude was the variable X, dispositional gratitude was the variable Y and seeking help, W, was the moderator (Figure 1).

## 2. Materials and Methods

### 2.1. Ethics Approval

This research project was authorized by the Bioethics Committee of the Institute of Psychology at the University of Szczecin (KB 13/2021, 20 May 2021) and the procedure was implemented in compliance with the Declaration of Helsinki.

### 2.2. Participants and Procedure

In total, 189 homeless persons participated in the study. Their mean age was *M* = 56.55 (*SD* = 12.39; range = 27–86). Most respondents were men (*n* = 119; 63%). As regards their level of education, 32% declared a primary school education, 39% a vocational school education, 24% a high school education and 5% a higher education. When asked about the length of time they were homeless, the participants responded in very different ways. Among them were those who became homeless in the month preceding the survey and also those who had lived in the shelter for 61 years. The mean duration of homelessness was *M* = 8.99 years (*SD* = 8.86). Among the most common causes of homelessness self-reported by the participants was: alcohol or drug abuse (42 respondents), being evicted (29), divorce (18), family disagreement (16), financial debts (16), domestic violence (9), own or family members’ illness (8), lack of work (7), failure to pay the rent (5), accident (4), prison (3), and gambling (2). Some of the participants pointed to several reasons and others refused to talk about the reasons for their predicaments. We did not have the opportunity to verify whether these causes were also related to mental disorders, although this cannot be excluded. It can be assumed that the real factors may also include some determinants that were not mentioned by the participants due to limited self-insight or shame.

The data collection procedure was carried out in nine centers where homeless people can stay overnight, have free meals, receive advice from a social worker, and consult a substance abuse counselor, therapist or psychologist: Caritas of the Archdiocese of Szczeciń-Kamień in Szczecin (31%), Accommodation of St. Brother Albert for homeless men in Gdańsk (27%), Accommodation of St. Brother Albert for homeless women in Wrocław (15%), Accommodation for Supporting the Needy “Przystań” for homeless men in Gdańsk (12%), Poviat Crisis Intervention Center in Słupia near Kępno (7%), “Street Church” Organization in Szczecin (3%), “New Beginning” Foundation in Szczecin (2%), Polish Red Cross–Regional branch in Kalisz (2%) and Religious Congregation of the Missionaries of Charity in Szczecin (1%). When asked whether the participants were looking for specific help from other people or institutions, a narrow majority responded in the affirmative (56%). The most frequently mentioned organization from which people sought help was the Municipal Family Assistance Center, a local government institution providing social assistance, operating in each commune. Homeless persons participated in the study through the “paper-and-pencil” form. To the question “What has been most difficult for you during the COVID-19 pandemic?” almost 50% of participants answered with isolation from people and loneliness. Other difficulties were related to the wearing of masks and the fear of infection and death.

### 2.3. Scale of Religious Attitude Intensity

The Scale of Religious Attitude Intensity developed by Prężyna and adapted by Śliwak and Bartczuk [96] measures the willingness of an individual to react positively or negatively toward a subject that has a religious character: God, Church understood as a religious institution, or the whole supernatural reality which can include faith in the action of grace or belief in angels, to name a few. Examples of statements relating to positive attitudes are: “Friendship with God is the greatest good that a person can achieve in life”; “The world without God is incomprehensible” and “Nothing happens in human life and human history without God’s will.” Examples of statements relating to negative attitudes are: “God is a fiction”; “People who accept supernatural reality are mindless” and “As science develops, all religions lose their raison d’être.” The scale contains 20 items, 10 of which are negatively formulated and need to be reversed. The participants were asked to rate their opinions on a 7–point Likert scale (1 = I strongly disagree; 2 = I disagree; 3 = I rather disagree; 4 = I can’t decide; 5 = I tend to agree; 6 = I agree; 7 = I strongly agree). The overall score was the sum of the points obtained by the person on the entire scale. A high score meant a high degree of intensity of religious attitude. The Scale of Religious Attitude Intensity has been corroborated in various former studies, showing its validity. For example, it correlated positively with the dimensions and the overall outcome of the Centrality of Religiosity Scale [97]. Moreover, in the previous analyses [98], the Cronbach α value was high, amounting to 0.95. In the current study, the reliability index was satisfactory and amounted to α = 0.81.

### 2.4. Gratitude Questionnaire

The Gratitude Questionnaire [99] adapted into Polish by Kossakowska and Kwiatek [100], assesses individual differences in the generalized tendency to experience gratitude. The questionnaire is a six-item self-report measure that reflects the four facets of gratitude: intensity, frequency, density, and duration. All of the items are rated on a seven-point scale (1 = strongly disagree; 2 = disagree; 3 = slightly disagree; 4 = neutral; 5 = slightly agree; 6 = agree; 7 = strongly agree). Items 3 (“When I look at the world, I don’t see much to be grateful for”) and 6 (“Long amounts of time can go by before I feel grateful to something or someone”) are reverse-scored. The remaining items are positively worded (e.g., “As I get older, I find myself more able to appreciate the people, events, and situations that have been part of my life history”). The scores range from 6 to 42, with a higher total meaning a higher level of gratitude. In the Polish research context, the Gratitude Questionnaire is well-known and usually shows the inner coherence at the level of Cronbach’s alpha starting from 0.67 [101], through 0.70 [102] to 0.78 [103]. The internal consistency of the whole scale was acceptable with α = 0.69.

### 2.5. Statistical Analysis

All statistical analyses were carried out using the SPSS software version 20 (IBM, Armonk, NY, USA). Descriptive statistics for religiosity and gratitude were calculated. The variables were tested under the assumptions of a normal distribution. We adopted the principle of values for skewness between ±2 and for kurtosis between ±5 [104] as acceptable for approximately normal distributed variables. Pearson’s correlation was used to check the association between intensity of religious attitude and dispositional gratitude.

The G*Power analysis program in version 3.1.9.4 [105] was used *a priori* to determine the appropriate sample size for this study. The higher power criteria of 0.95 and a significance criterion α of 0.05 for the t-test to establish a small effect size (*r* = 0.25) were chosen. The G*Power analysis showed that a minimum sample size for the appointed criteria would require at least 164 respondents. The choice of a small effect was dictated by the fact that in many studies the correlation between various dimensions of religiosity and gratitude is relatively low and does not exceed or slightly exceeds *r* = 0.30. For example, Kraus et al. [82] showed that gratitude is positively associated with private devotion, religious salience and efficacy between *r* = 0.18** and *r* = 0.19**. Yost-Dubrow and Dunham [106] reported a modest positive correlation between both variables. Watkins et al. [107] observed a positive relationship between gratitude and intrinsic religiosity (*r* = 0.32**) and a negative relationship between gratitude and extrinsic religiosity (*r* = −0.28*).

A linear regression model was used to adjust for potential confounders including sex, age, and the length of time of homelessness. There is some evidence that women and men differ with respect to their attitudes toward religiosity, gratitude, and homelessness. Many authors consistently find that on average women score higher on the measures of religious dimensions compared to men [108,109,110]. Likewise, women generally tend to report higher feelings of gratitude and express more gratitude than men [111,112]. In respect to homelessness, the “typical” profile of a homeless person in most countries is a man between 30 and 50 years of age [113,114]. In Poland, this is also the case [115]. With respect to age, older people are commonly more religious in comparison to younger adults [116,117,118]. With respect to gratitude, research yields mixed evidence [119]. In some studies, gratitude remains relatively stable across a life span [119,120]. However, other studies emphasize that there are some changes in gratitude in a life span [121]. For example, other authors [119,122] showed that younger people express gratitude differently to older people, showing lower levels of expressing gratitude than seniors.

The moderating effect was tested using the PROCESS macro (version 3.2) and Model no. 1 [123].

## 3. Results

### 3.1. Preliminary Analyses

Religious attitude intensity and dispositional gratitude were screened for skewness and kurtosis to assess the normality of the scales’ distribution. None of them exceeded the values of ±2 for skewness and ±5 for kurtosis (Table 1).

### 3.2. Correlations

The results of the linear relationship between paired variables using Pearson’s *r* coefficient presented a statistically significant positive correlation between religious attitude intensity and dispositional gratitude *r* = 0.326***, *p* = 0.001. Thus, H1 was confirmed.

### 3.3. Multicollinearity and Confounding Variables

The multiple linear regression showed that there was no problem with collinearity for the sample’s data since the range of VIF values was between 1.010 and 1.052, and the tolerance values were between 0.950 and 0.990. The Mahalanobis distance indicated no presence of outliers. Cook’s values were well below 1, with the range between 0.000 and 0.060. The results showed that sex (*β* = −0.062; *t* = −0.864; *p* = 0.389), age (*β* = 0.066; *t* = 0.925; *p* = 0.356), and the length of time of homelessness (*β* = −0.061; *t* = −0.852; *p* = 0.396) were not relevant confounders since they accounted for only 1.3% of the variance (*R^2^* = 0.013; *β* = 0.112; *t* = 0.781; *p* = 0.506). The intensity of religious attitude explained an additional 9% of the variance.

### 3.4. Moderation

The results of the moderation analysis obtained through the procedure of the bias-corrected bootstrapping method [123] with 95% for the confidence interval from 5000 resamples showed good fit to the data (*F*(3, 185) = 8.81, *p* < 0.001). The model explained 12% of the variance of the dispositional gratitude (*R*^2^ = 0.12).

**Figure 2:**
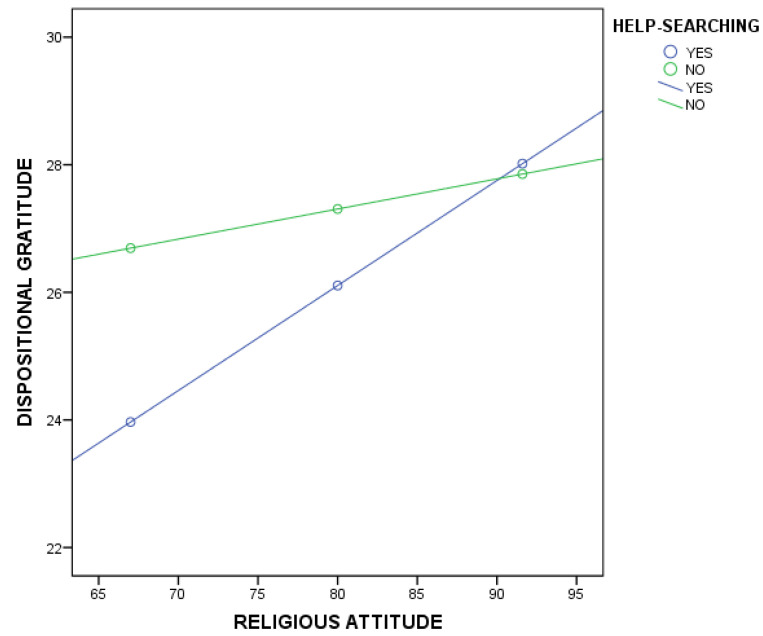
Plot of the slope analysis for the moderating effect of seeking help in the relationship of religiosity and gratitude among homeless people.

The regression between religious attitude intensity and dispositional gratitude was statistically significant with the values of *b* = 0.28, *t*(185) = 3.45, *p* < 0.001, 95% CI [0.1208;0.4430]. Moreover, the regression for the total interaction coefficient of the model tested was statistically significant with *b* = −0.12, *t*(185) = −2.22, *p* < 0.05, 95% CI [−0.2217;−0.0131]. The outcomes (Figure 2) displayed that the moderation effect was significant only for the condition of seeking help (YES) with *b* = 0.16, *t*(185) = 4.57, *p* < 0.001, 95% CI [0.0936;0.2356] and not for the condition of not seeking help (NO) with *b* = 0.04, *t*(185) = 1.21, *p* = 0.22, 95% CI [−0.0291;0.1236].

Therefore, it can be assumed that the relationship between religious attitude intensity and dispositional gratitude is stronger when homeless persons seek specific help from other people or institutions compared to when they do not look for assistance.

## 4. Discussion

The first aim of the current study was to provide support for the hypothesis that religious attitude positively correlates with dispositional gratitude among homeless persons (H1). The second aim was to answer the hypothesis that the level of gratitude resulting from intensity of religious attitude is significantly different at different levels of seeking help by homeless persons (H2). Both hypotheses were confirmed.

Regarding the first hypothesis, although it might be expected that people with a hard life experience [124] have scarce occasions to be grateful, they actually still find reasons to be grateful, such as simply being alive or being sheltered. Moreover, although society is not accustomed to think about the function of religion in the lives of homeless persons [125], it seems that sometimes these people find their strength in God or divinity [126]. Smith-Barusch [90] reported that the dominant topics among most homeless persons are religious experiences and expressions of gratitude. In narratives from the streets, Phillips [127] described examples of homeless people who appreciated the smallest gifts which reduced their levels of anxiety. These persons acknowledged their vulnerability and spoke of being highly grateful. Jindra et al. [63] drew attention to the fact that both religion and gratitude are integral components of how people who struggle deal with their problems. This is because both religiosity and gratitude help in self-transcending and opening oneself to the divine or to other people. Furthermore, an important aspect that emerged from this study is that the level of religiosity and gratitude of the homeless, although lower than that normally found in the general population, was higher than the average attainable on both scales. Such a result may mean that, despite the difficulties of being deprived of a home and family relationships, homeless people have a certain intensity of religious attitudes and disposition for gratitude that help them cope with their life conditions.

With respect to the second hypothesis, our findings bring some new insights into the knowledge about the relationship between religiosity and gratitude, showing that this association can be more intense when homeless people seek help than when they do not look for assistance. Although there are not specific studies on this topic, we can assume that religiosity, gratitude, and asking for help share some characteristics. First, coping is the first common denominator of all three variables. Help-seeking in distressing or stigmatized circumstances is recognized as an adaptive form of coping and a protective factor [94,128]. Similarly, religiosity [129,130] and gratitude [131] are widely recognized as those human experiences that are associated with more active coping strategies. Second, the sense of reliance is another aspect that connects religiosity, gratitude, and seeking help. Religious reality, as well as gratitude and seeking help remind us that people live in a world of mutual connections in which they not only give, but also receive. Therefore, the homeless who look for help must overcome the stigma and shame related to their precarious conditions [132]. They also demonstrate their ability to deal with problems despite difficult circumstances. Such behavior may indicate that homeless people who ask for help do not want to be left alone, count on support of the divine or others, and may indicate that they depend on them. These individuals who engage in looking for assistance may do so because they draw strength from a religious foundation. Third, the reciprocal nature of the interpersonal relationships that connect religiosity, gratitude, and seeking help requires admitting dependence and vulnerability. Asking for help involves some level of vulnerability/sensitivity [133] which, in turn, is needed in experiencing and expressing gratitude and religiosity. In fact, Solomon [134], p. V) acknowledges that being grateful “involves an admission of our vulnerability and our dependence on other people.” At the same time, awareness of vulnerability is connected with taking care of each other [135]. Therefore, the association between religious attitude and gratitude may be more intense when homeless persons admit to being in need and find in themselves the strength to seek help. Conversely, those homeless who do not look for help may think that they can manage on their own, or do not want to depend on others or change anything in their lives. Nadler ([136], p. 382) sees in the decision to not seek help resignation and the choice to “live with the problem.” In such a situation, there is little room for being reliant on Another–understood as God or a higher power–or on other people.

## 5. Limitations

It is important to address some limitations of our study. The first is the lack of information on the churchgoing practices and the affiliation of the homeless who we met to a particular religion and the degree to which they identified with a given religion or ideology. Moreover, most of the literature on the relationship between religiosity and health or well-being is conducted from a Christian perspective. It is very likely that among the surveyed homeless, most of them were in some way related to the Christian faith due to its leading role in Poland and to the aid institutions run by the Catholic Church, the help of which these homeless people benefited from. Therefore, this aspect does not allow strong conclusions about the relation between religiosity and gratitude in general but only for the relation between Christian beliefs and grateful disposition. However, even if the homeless participants adhered to a faith other than Christianity, it can still be assumed that their belonging to any other faith may strengthen their gratitude. This is in line with previous research showing that adherence to both Abrahamic faiths [137,138,139] and Eastern religions can be related to gratitude [51,140,141]. Another aspect worth mentioning is testing for specific addictions or ascertaining the level of mental health. In future studies, taking these factors into account would be important for measuring potential confounding variables.

## 6. Conclusions

The results of this study indicate that the effort to ask for assistance is not without significance for the relationship between the religiosity and gratitude of homeless persons. The outcomes also show that homeless people, overcoming their limitations by actively asking for help, can strengthen their bonds with God (faith, religiosity) and with others (dispositional gratitude).

## Figures and Tables

**Table 1:** Descriptive statistics for the Scale of Religious Attitude Intensity and the Gratitude Questionnaire (N = 189).

Scales	*M*	*SD*	Skewness	Kurtosis
Religious Attitude Intensity	78.26	18.54	−0.827	−0.731
Dispositional Gratitude	26.28	7.02	2.359	0.799

## Data Availability

The datasets used during this study are available from the corresponding author.
